# Fever of unknown origin as the first presentation of candida vertebral osteomyelitis: A case report

**DOI:** 10.1002/ccr3.5582

**Published:** 2022-03-23

**Authors:** Athena Farahzadi, Habibollah Mahmoodzadeh

**Affiliations:** ^1^ Division of Surgical Oncology Cancer institute Tehran University of Medical Sciences Tehran Iran

**Keywords:** candida osteomyelitis, vertebral osteomyelitis

## Abstract

Candida osteomyelitis is a rare disorder; however, its incidence has drastically risen especially during the last 3 decades. Diagnosis is usually delayed due to nonspecific symptoms. Thus, its management would not be performed at the proper time, which leads to increased morbidity. The optimal management of candida osteomyelitis is not clearly defined but most recommendations advise prolonged antifungal therapy in addition to surgery. Here, we report candida osteomyelitis in a patient with a history of pancreatic adenocarcinoma that presented with prolonged FUO as her first presentation. Fortunately, her symptoms completely resolved after prolonged medical therapy despite delay diagnosis due to her unusual presentation. To the best of our knowledge, it is the first report of candida vertebra osteomyelitis that presents with prolonged FUO as the first presentation.

## 1 INTRODUCTION

Candidemia, previously considered transient and harmless in most cases, is now known to be frequently complicated by deep‐seated infections in various parts of the body like eye, kidney, liver, skin, and heart Bone and joint infections are rare secondary foci.^1^

Fungal spondylodiscitis is rare and consists of 5% of all cases of spondylodiscitis, candida species are the most frequent agent. Fungal Monilia psilosis osteomyelitis was first described by Conner in 1928. Keating reported four other patients with Monilia osteomyelitis involving long bone sites in 1932. The first report of Candida osteomyelitis came back in 1970.^2^, ^3^

The incidence of invasive candida infection has risen dramatically in the last 30 years due to an increased number of immunocompromised patients, invasive procedures, and the use of broad‐spectrum antibiotics although it is still extremely rare.^4^

The unusual presentation of candida vertebral osteomyelitis (CVO) makes early recognition of the disease difficult therefore depriving the patient of timely intervention and can cause significant morbidity. The treatment strategies of invasive candida infections have evolved, it consists of medical antifungal therapy for a long time, and in most cases, surgery is a part of the main treatment. The Infectious Diseases Society of America (IDSA) recommendations for CVO include surgical debridement and an initial course of amphotericin B for 2–3 weeks, followed by fluconazole for a total duration of 6–12 months.^1^, ^3^, ^5^ Here, we report a patient with a history of pancreatic cancer who was diagnosed with CVO several months after her operation that consisted of distal pancreatectomy, splenectomy, and adrenalectomy. She received prolonged antifungal treatment and her symptoms were completely resolved without surgery.

## 2 CASE PRESENTATION

A 70‐year‐old woman with incidental findings of distal pancreatic mass during screening workup came to our clinic for further evaluation. EUS and FNA were performed. The pathology specimen was reported as adenocarcinoma. Her past medical history was uneventful except her recent diagnosis of pancreatic cancer. Distal pancreatectomy, splenectomy, and adrenalectomy were performed due to vascular involvement. Port was placed in the right internal jugular vein at the time of surgery because of a lack of proper peripheral vascular access. On the fifth postoperative day, she became febrile. Abdominal ultrasonography showed peripancreatic collection. A pigtail catheter was inserted under ultrasonography guidance, the collection was drained, and sent for a culture that turned out to be negative. Antibiotics were continued, she had a persistent low‐grade fever.

**FIGURE 1:**
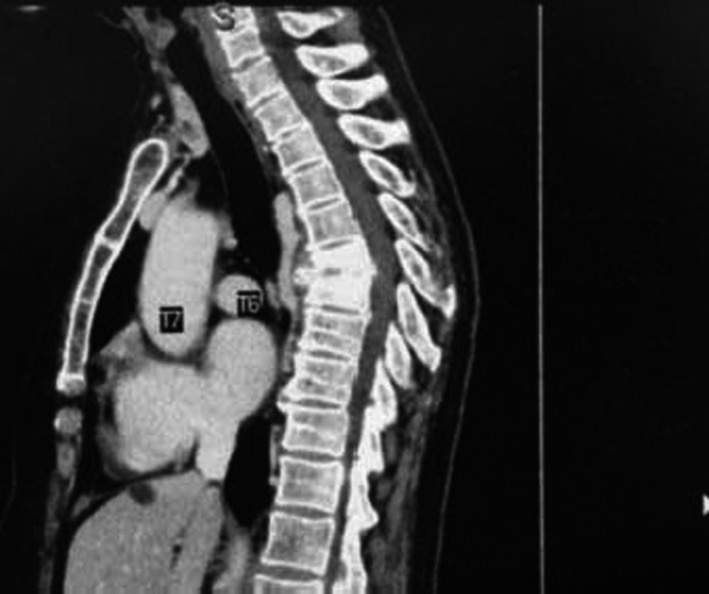
Thoracic CT scan, sagittal view, shows destruction of the body of T6, T7 vertebrae

**FIGURE 2:**
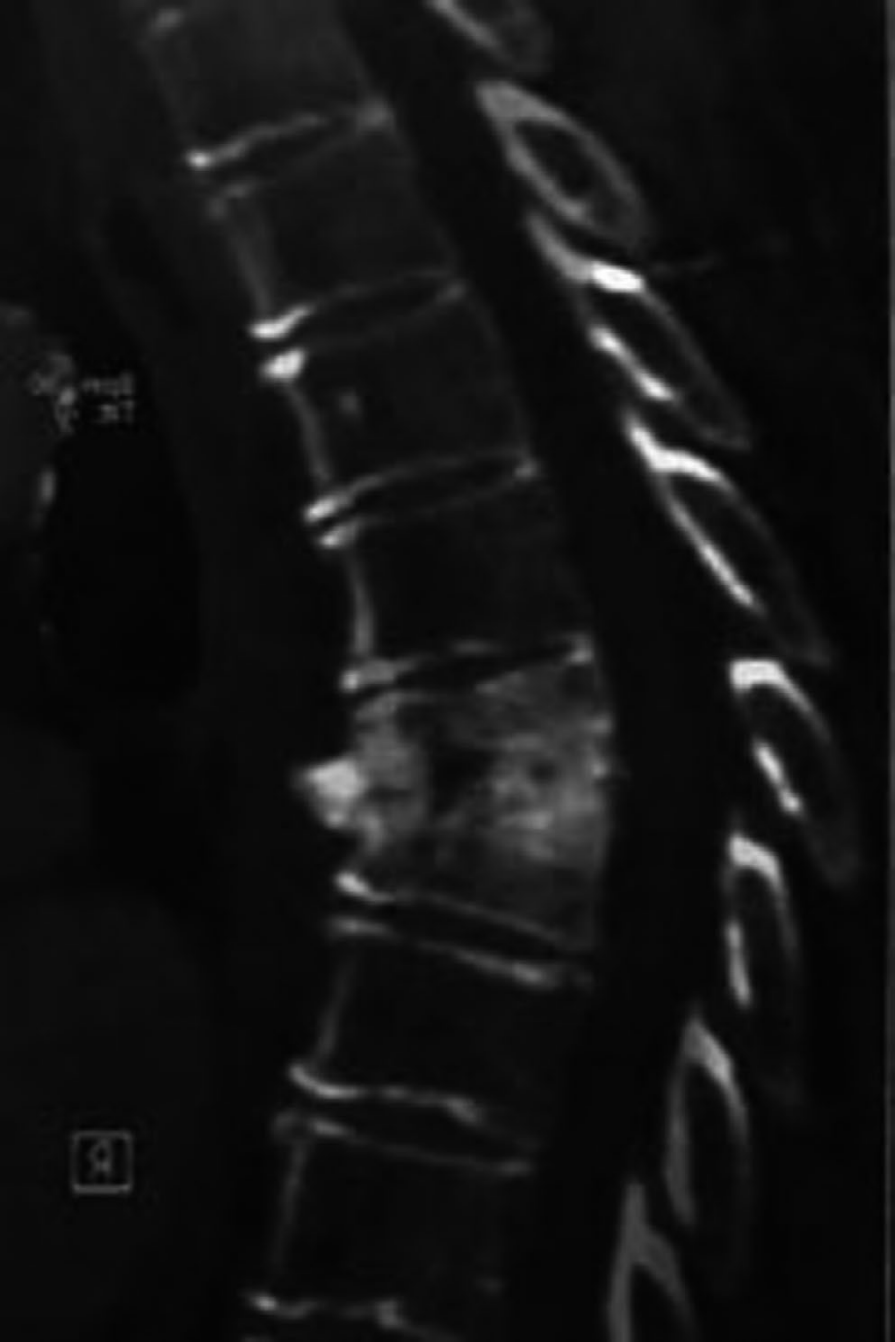
Thoracic sagittal T1‐weighted MRI image shows destruction and collapsed of the body of T6, T7 vertebrae

**FIGURE 3:**
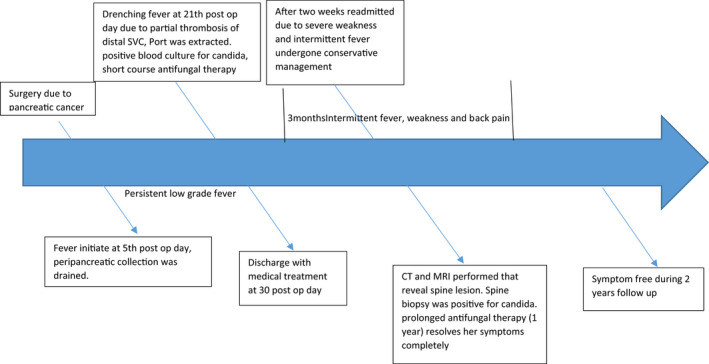
Patient’s past medical history, clinical management, and treatment

On the 21st postoperative day, she had high‐grade drenching fever with respiratory distress, chest CT angiography showed partial thrombosis of the distal part of SVC containing air bubbles, suspicious for the infected clot. The port was brought out and sent for a culture that turned out positive twice for candida, simultaneous blood culture was negative three times. The abdominal CT scan was normal at that time. Laboratory testing gave generally nonspecific results. Full sepsis workup investigation turned out to be non‐diagnostic. Physical examinations including the neurologic examination revealed no abnormality. Antifungal therapy (caspofungin) was added to her regime although the positive candida culture of the port was regarded insignificant by our infectious disease specialist. She was discharged with caspofungin in good physical condition after 30 days, she was not febrile anymore. Two weeks later, she suffered from severe weakness, back pain, and low‐grade fever. The patient was readmitted to the hospital, and conservative management was done. The abdominal CT scan seems to be normal. After three months, another thoracoabdominal CT scan was performed due to persistent weakness, back pain, and on and off low‐grade fever that showed destruction of two thoracic vertebral bodies (T6,7) (Figure 1). The neurologic examination was normal. She had no difficulty with ambulation apart from her severe physical weakness. Thoracic MRI has been performed that revealed destruction of T6, T7 (Figure 2). Biopsy was taken from the affected vertebral body, candida was cultured, and diagnosis of CVO was made. Fluconazole was initiated with a dose of 400mg daily, her persistent fever was subsiding gradually. She continued antifungal therapy for one year. Now she has normal neurologic examination, with no back pain; however, she complained of occasional pain on the left side of lumbar vertebrae (Figure 3).

## 3 DISCUSSION

Invasive fungal infections including nosocomial bloodstream infections due to Candida species have increased significantly over the last years.^3^ Despite the increase in the frequency of fungemia, infection by Candida. spp is also a rare cause of spinal infection.^6^CVO typically occurred in immunocompromised patients or in intravenous drug users previously. Increased antibiotic use, placement of a central venous catheter, chemotherapy, steroid use or other immunosuppression, surgery, placement of a urinary catheter, underlying malignancy, diabetes, alcohol abuse, parenteral nutrition, hemodialysis, burns, neutropenia, and the isolation of Candida organisms from >2 body sites are reported in the literature as risk factors for candidemia. These risk factors can alter the patients’ natural flora, outreach the risk for candidemia from an intravascular device, or cause some level of immunosuppression.

In 1998, Anderamahr reported a case of lumbar spondylitis due to Candida and then after reviewing 31 adult cases with vertebral osteomyelitis from the literature. In our review of the published English literature through PubMed for candida spondylodiscitis, about 200 cases of candida spondylodiscitis were found mostly reported in the last two decades and most of them were in immunocompromised patients or IV drug users.^2^, ^7^

CVO may occur either simultaneously or several months after an episode of verified or suspected candidemia. In patients with suspected candidemia which is not verified by culture, risk factors for candidemia can be noticed in two‐thirds of them.^4^ CVO can enlace any bone, however, the axial skeleton is most involved in adults. According to some studies, an antifungal regimen enough to clear the episode of fungemia may not prevent the occurrence of this late complication.^8^ Blood culture results are reported to be positive in only 50% of cases of documented disseminated candidiasis.^9^, ^10^ Therefore, cases with prior candidemia may not be detected only through blood culture.^4^ Because CVO is regarded as a complication of an episode of candidemia, a high index of suspicion must be retained for patients with a history of candidemia who present with back pain.^11^

Early diagnosis of CVO is difficult. The lapse in diagnosis between the onsets of symptoms to the recognition of CVO has been reported as ranging from 1 month to several years.^12^, ^13^ With an average of 3.3 months.^2^ This can be due to delay in presentation, investigation, vogue symptoms, the insidious course of the disease, the lack of a specific diagnostic test, and the reluctance of clinicians to see Candida spp. as a true pathogen.^8^, ^14^

The most common presentation of the disease is back pain mostly in the lower thoracic to the lumbosacral spine. Only one‐third of patients had a fever at the presentation. In our research, we do not find a case of CVO who presents with prolonged FUO as the first presentation like our case. About 20% of patients had neurological deficits.^14^, ^15^

Results of laboratory tests except microbiological tests were nonspecific; they frequently consisted of an elevated ESR but a normal WBC count.^14^ Therefore, clinical and standard laboratory findings are incomplete to precisely diagnose CVO. A high clinical index of suspicion, with proper radiographic studies followed by verifying with microbiological tests, helps to diagnose the disease appropriately. Erosive and destructive vertebral changes may be seen in plain radiography, but with weeks to months’ delay.^16^ There is no characteristic CT finding for CVO; however, CT scan can show earlier bony changes and can appraise the presence of paravertebral or spinal canal extension, too.^17^ Tumoral or tumor‐like spine lesions, metabolic and genetic disorder, avascular necrosis, infection, idiopathic osteolysis disease like Gorham syndrome can be regarded as differential diagnosis of imaging findings. MRI and PET‐CT scan are more sensitive and can be used in early stages. The culture of a biopsy specimen is required for the definitive microbiological diagnosis of CVO. Multiple needle biopsy specimens should be taken from involved vertebral bodies, intervening discs, and paravertebral soft tissues, to make a definite diagnosis. If the results for primary needle biopsy specimens are negative, the procedure should be repeated. If a second set still reports negatively, then performing an open biopsy should be considered seriously, because empirical therapies for bacterial, mycobacterial, or fungal osteomyelitis are vastly different and associated with toxicities that require close monitoring.^4^, ^14^ Candida PCR assays of biopsy specimens are more sensitive and rapid than culture. They do have value as complementary tests. But they are expensive, and culture is still the gold standard.^18^

Patients with CVO have a good prognosis and most of them without significant comorbidities were clinically completely cured, similar to patients with bacterial vertebral osteomyelitis.^15^ The cure rate for cases with CVO is reported to be about 85%. But without treatment, the disease progresses and leads to vertebral destruction and spinal cord and neural compression. As soon as osteomyelitis is dubious, investigations with proper imaging and percutaneous biopsy should be done followed by medical therapy in order to halt the advancement of bony destruction and therefore barricade the need for surgery. Surgical debridement, fusion, and stabilization combined with medical therapy can successfully extricate the infection and resolve the neurological deficits if vertebral collapse and spinal cord compression happen. In our case, despite the destruction of lumbar bodies, the patient was fully recovered without any surgery with prolonged antifungal therapy.^14^Little is known about the optimized management of candida spondylodiscitis, yet fluconazole seems to be effective in most cases and prolonged treatment of several months seems to be vital as in our case.^1^

In the case that we present here, it seems that history of pancreatic cancer, prolonged antibiotic use, plus central venous catheterization susceptible our patient to candidemia. It manifests with prolonged FUO, and after few months low back pain added to her symptoms that finally led to definite diagnosis and proper management.

We hope that case reports such as this one help to increase the experience in the management of this rare disease.

## CONFLICTS OF INTEREST

There are no conflicts of interest.

## AUTHOR CONTRIBUTIONS

All the authors made significant contribution to the study and have read and approved the final version of the manuscript.

## CONSENT

Written informed consent was obtained from the patient to publish this report in accordance with the journal's patient consent policy.

## Data Availability

The data that support the findings of this study are available on request from the corresponding author. The data are not publicly available due to privacy or ethical restrictions.
